# Newly Synthesized Thymol Derivative and Its Effect on Colorectal Cancer Cells

**DOI:** 10.3390/molecules27092622

**Published:** 2022-04-19

**Authors:** Michaela Blažíčková, Jaroslav Blaško, Róbert Kubinec, Katarína Kozics

**Affiliations:** 1 Cancer Research Institute, Biomedical Research Center, Slovak Academy of Sciences, Dúbravská cesta 9, 84505 Bratislava, Slovakia; michaela.blazickova@savba.sk; 2 Department of Analytical Chemistry, Faculty of Natural Sciences, Comenius University in Bratislava, Mlynská dolina, Ilkovičova 6, 84215 Bratislava, Slovakia; jaroslav.blasko@uniba.sk (J.B.); robert.kubinec@uniba.sk (R.K.)

**Keywords:** thymol, acetic acid thymol ester, thymol β-D-glucoside, cytotoxicity, genotoxicity, ROS, colorectal cancer

## Abstract

Thymol affects various types of tumor cell lines, including colorectal cancer cells. However, the hydrophobic properties of thymol prevent its wider use. Therefore, new derivatives (acetic acid thymol ester, thymol β-D-glucoside) have been synthesized with respect to hydrophilic properties. The cytotoxic effect of the new derivatives on the colorectal cancer cell lines HT-29 and HCT-116 was assessed via MTT assay. The genotoxic effect was determined by comet assay and micronucleus analysis. ROS production was evaluated using ROS-Glo™ H_2_O_2_ Assay. We confirmed that one of the thymol derivatives (acetic acid thymol ester) has the potential to have a cyto/genotoxic effect on colorectal cancer cells, even at much lower (IC_50_~0.08 μg/mL) concentrations than standard thymol (IC_50_~60 μg/mL) after 24 h of treatment. On the other side, the genotoxic effect of the second studied derivative—thymol β-D-glucoside was observed at a concentration of about 1000 μg/mL. The antiproliferative effect of studied derivatives of thymol on the colorectal cancer cell lines was found to be both dose- and time-dependent at 100 h. Moreover, thymol derivative-treated cells did not show any significantly increased rate of micronuclei formation. New derivatives of thymol significantly increased ROS production too. The results confirmed that the effect of the derivative on tumor cells depends on its chemical structure, but further detailed research is needed. However, thymol and its derivatives have great potential in the prevention and treatment of colorectal cancer, which remains one of the most common cancers in the world.

## 1. Introduction

Colon cancer (CRC) is the third most common cancer in the world, after lung and breast cancer. In 2018, an estimated 1.8 million new cases of colon cancer were identified. In terms of mortality, CRC is in second place worldwide. The most common incidence is recorded in Central Europe, Australia, and North America [1]. However, a sufficiently effective method of treatment and prevention has not yet been developed. At present, treatment is most often based on invasive surgery, radiotherapy, or chemotherapy. The cytotoxic drugs used often have many side effects. The application of natural compounds or their derivatives may be safer and may represent an effective means of increasing the effectiveness of treatment [2].

Plants of the *Lamiaceae* family are characterized by a high content of essential oils. *Thymus vulgaris*, a herb that occurs naturally in areas of southern Europe, has a high content of thymol, terpenoids, flavonoids, glycosides, and phenolic acids [3,4]. The content of thymol depends on several factors: environmental or genetic, but also on the chemotype of *Thymus vulgaris*. There are different chemotypes according to the predominant monoterpene in the essential oil, for example, the thymol or carvacrol chemotype [5,6]. Thymol, chemically known as 2-isopropyl-5-methylphenol, is a white crystalline substance with a characteristic aroma and is defined as safe with negligible toxicity by the United States Food and Drug Administration (US FDA) [7]. Various studies have confirmed its therapeutic effects on the human body. It has antioxidant, antifungal, or antibacterial effects [8]. It also effectively affects tumor cell lines, including colorectal cancer cells [9,10]. In the colorectal cancer cell line HT-29, thymol affects the PI3K/AKT and ERK signaling pathways, thereby inhibiting cell migration [9]. Another study observed activation of the Wnt/β catenin signaling pathway following the application of thymol to the HCT-116 cell line. In vivo, the presumption of reduction in colorectal cancer growth by the action of thymol was confirmed at the level of the action of doxorubicin [11]. The solubility of thymol in water is low, hence its penetration into the cell and its application in practice are limited [12,13]. Therefore, new thymol derivatives have been synthesized, which are expected to be more efficient due to better cell penetration. In our study, we evaluated the effect of these derivatives on HT-29 and HCT-116 colorectal carcinoma cell lines. We determined the cytotoxic and genotoxic effects, the effect on cell proliferation, micronucleus formation, and ROS generation. Thymol derivatives could represent a more effective approach to the prevention and treatment of colon tumors.

## 2. Results

### 2.1. Proliferation Activity

**Figure 1:**
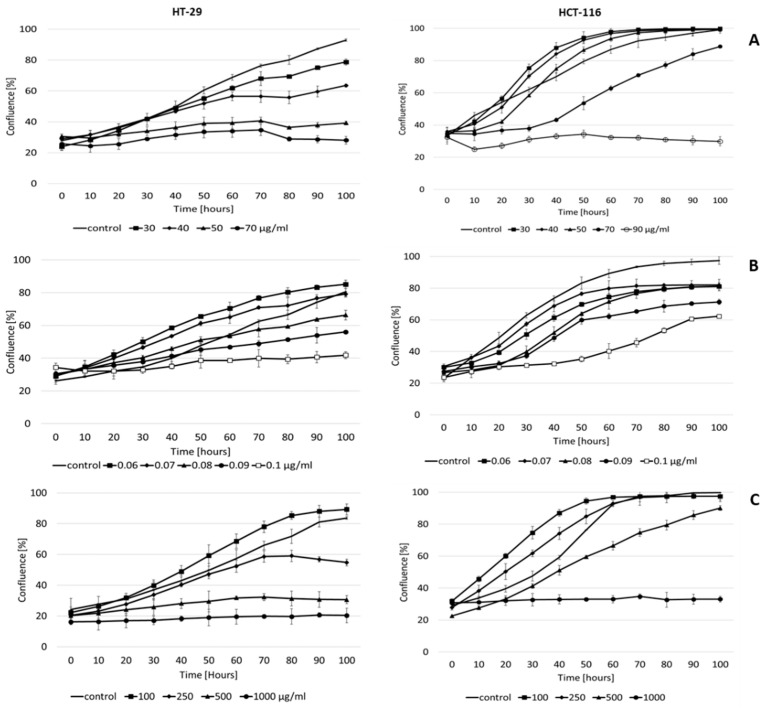
Effect of thymol-standard (**A**) and its derivatives DT1 (**B**) and DT2 (**C**) on the proliferation of cell lines HT-29 and HCT-116 during 100 h. Data are represented by means ± SD of three independent experiments.

For each of the tested substances, we observed a concentration-dependent slowing of the proliferation of colorectal tumor cell lines HT-29 and HCT-116. In general, the HCT-116 cell line showed greater resistance to all test substances. There was a significant decrease in proliferation with the standard thymol only at the higher concentration, in contrast to the HT-29 cell line, where we observed reduced confluence to approximately 40% at a concentration of 50 μg/mL (Figure 1A).

Low concentrations of thymol derivatives promoted proliferation. However, higher concentrations significantly limited cell growth. DT1 significantly reduced confluence to 40% at a concentration of 0.1 μg/mL (Figure 1B). We observed a more profound effect in DT2 when in both cell lines the confluence was reduced below 40% at the highest tested concentration of 1000 μg/mL and there was no significant change in cell growth throughout the experiment (100 h). We also observed the same effect at a concentration of 500 μg/mL in the HT-29 cell line (Figure 1C).

The results suggest that DT2 acts more efficiently on cell proliferation of both cell lines, but at concentrations several orders of magnitude higher compared to DT1.

### 2.2. Cytotoxicity Effects

**Figure 2:**
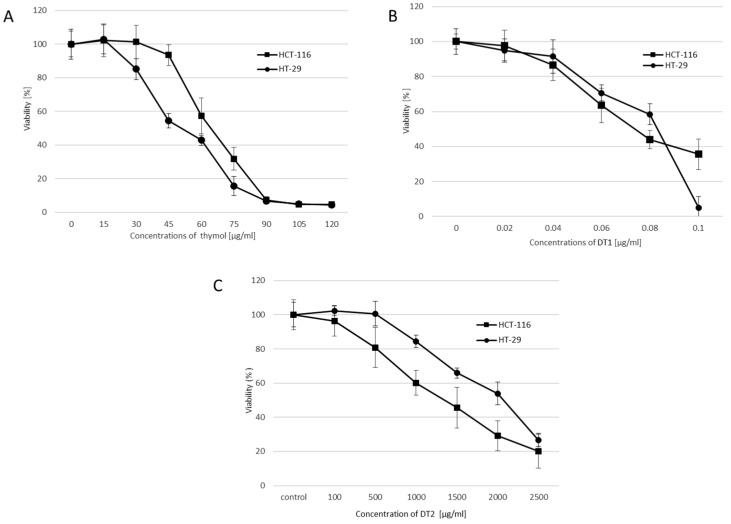
Cytotoxic effect of thymol-standard (**A**), DT1 (**B**), and DT2 (**C**) on tumor cell lines HT-29 and HCT-119 after 24 h of treatment. Data are represented by means ± SD of three independent experiments.

Cytotoxicity effects/changes in the viability of studied compounds on colorectal carcinoma cell lines HCT-116 and HT-29 were evaluated by the MTT assay. The results are summarized in Figure 2A–C. The curves represent the viability of cells after 24 h treatment with individual derivatives T, DT1, and DT2. The reduction in cell viability was directly dependent on the applied concentration of the tested substance. IC_50_ value (median inhibitory concentrations that cause approximately 50% cell death) for the standard in the HT-29 cell line is ~52 μg/mL and for the HCT-116 cell line is ~65 μg/mL (Figure 2A). Derivatives DT1 exhibited the highest cytotoxic activity (IC_50_~0.08 μg/mL) in both studied cell lines (Figure 2B). On the other hand, DT2 had a higher value of IC_50_ (for HT-29~2100 μg/mL and for HCT-116~1300 μg/mL) in comparison to the standard thymol (Figure 2C). Further studies aimed at the genotoxic effects of studied compounds were assessed at IC~_10–60_.

### 2.3. DNA-Damaging Effects

The level of DNA strand breaks induced by derivatives of thymol and standard thymol was determined by the comet assay in HT-29 and HCT-116 cells. For the induction of DNA single-strand breaks in studied cells, H_2_O_2_ at a concentration of 500 µM was selected and used as a positive control. The higher concentration of H_2_O_2_ was used as the colorectal cancer cells showed higher resistance to oxidative agents; 500 μmol/L of H_2_O_2_ induced strong DNA damage corresponding to about 50% of DNA in the tail.

**Figure 3:**
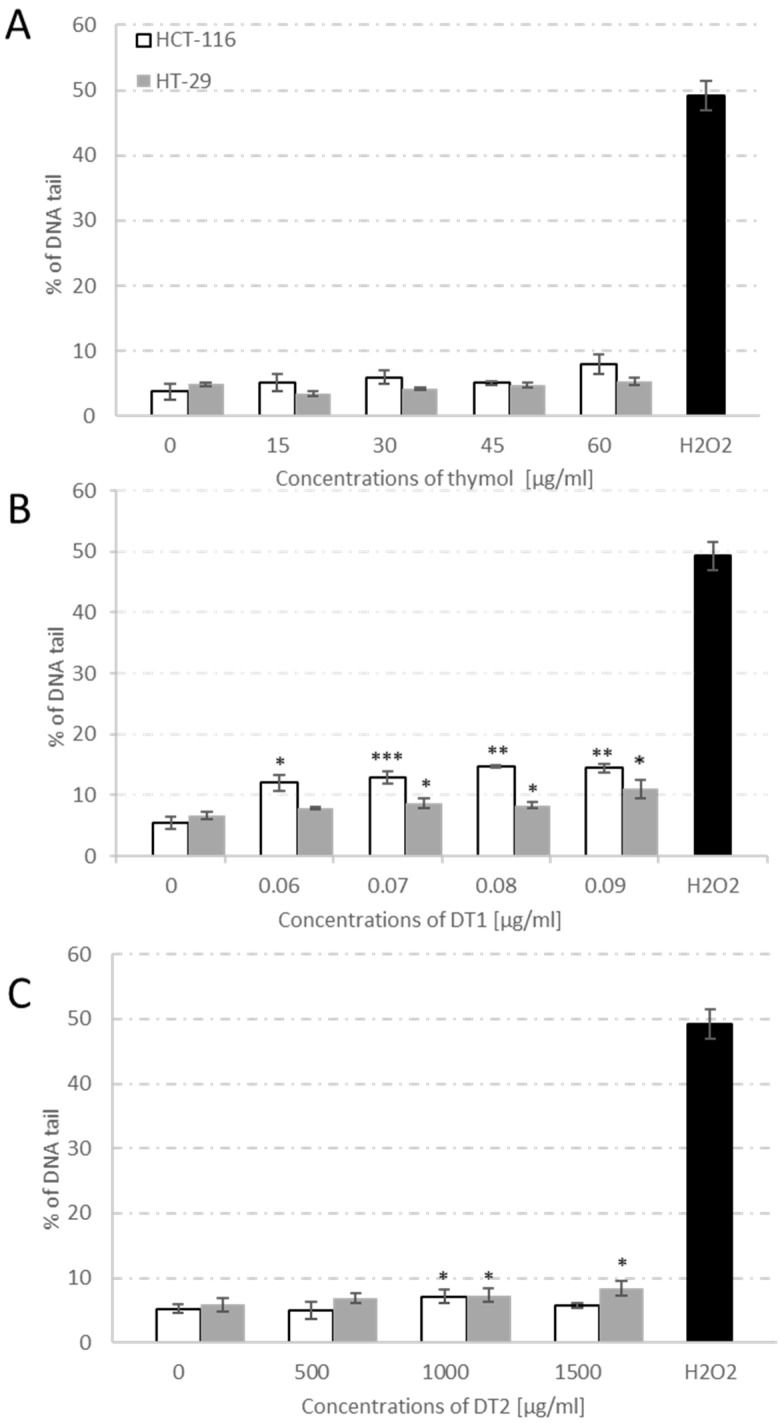
The levels of DNA single-strand breaks (% of tail DNA) in HT-29 and HCT-116 cells after the exposure to thymol (**A**), DT1 (**B**), and DT2 (**C**) for 24 h. Positive control-hydrogen peroxide (500 μmol/l). Data represent the means ± SD of three independent experiments. * *p* < 0.05; ** *p* < 0.01; *** *p* < 0.001 indicate statistically significant differences compared to the control (Student’s *t*-test).

Standard T did not induce DNA damage compared with untreated control cells in both cell lines. However, DT1 significantly increased the level of DNA damage in a concentration-dependent manner for the HCT-116 cell line, while for the HT-29 cell line this increase was observed from the concentration of 0.07 μg/mL (Figure 3B). DT2 significantly increased DNA damage at non-cytotoxic concentrations from 1000 μg/mL in the HT-29 cell line, while for the HCT-116 cell line only 1000 μg/mL.

### 2.4. Formation of Micronuclei

**Figure 4:**
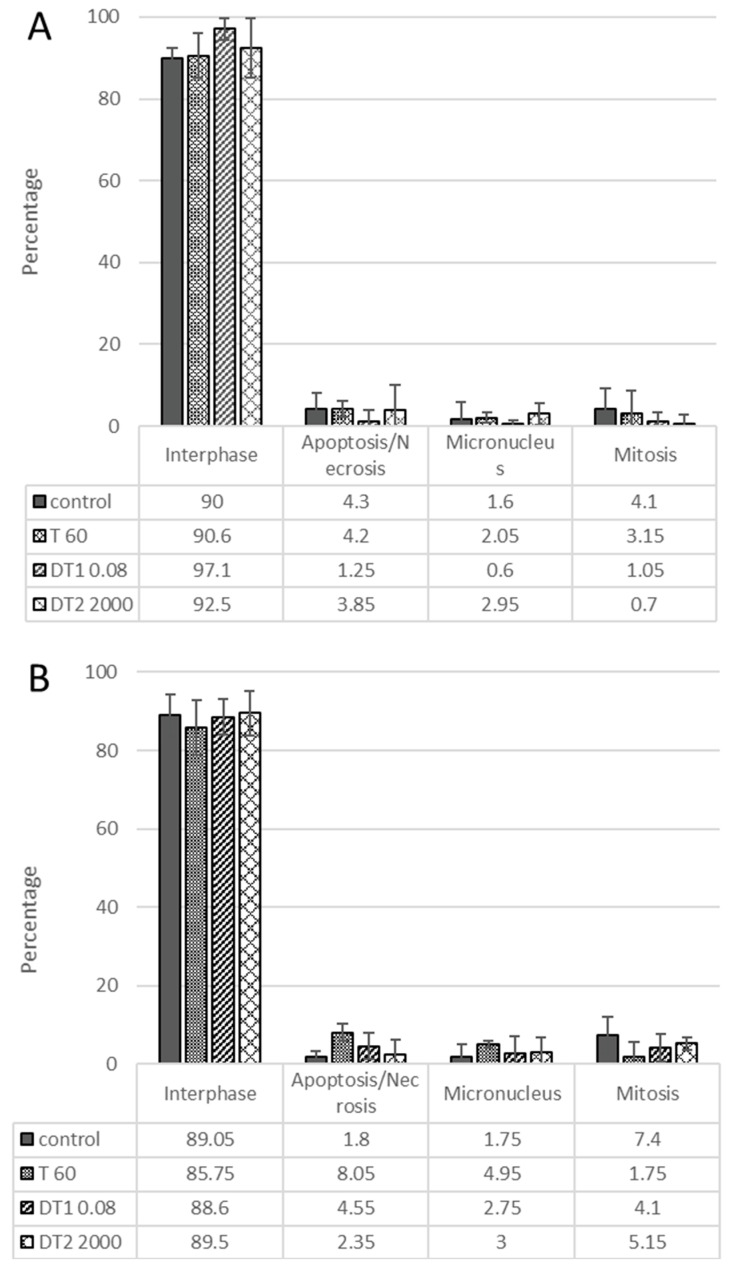
The effect of thymol-standard and newly synthesized thymol derivatives (DT1/DT2) on the formation of micronuclei on the HT-29 (**A**) and HCT-116 (**B**) cell lines after 24 h. Data represent the means ± SD of three independent experiments.

We also tested thymol derivatives for the formation of micronuclei. Thymol-standard did not show an increased rate of micronuclei formation at any of the concentrations tested. We also did not notice a significant increase in apoptotic or necrotic cells. We observed the same results for all other tested derivatives for HT-29 (Figure 4A) and HCT-116 cell lines (Figure 4B).

### 2.5. ROS Production

The effect of the studied compound on ROS production was evaluated at different concentrations (10–60 μg/mL for T, 0.01–0.08 μg/mL for DT1, 500–2000 μg/mL for DT2 in HT-29 and HCT-116 cells using ROS-Glo™ H_2_O_2_ Assay. The results for the HT-29 cell line indicate an increase in the luminescence intensity at 0.08 μg/mL (~380%) for DT1, while in HCT-116 cells, increased ROS production was only 151% (Table 1) in comparison to the untreated control cells. These concentrations actually induced cytotoxic and genotoxic effects on cells. Derivative DT2 significantly increased ROS production at a concentration of 1500 μg/mL, about 210% for both cell lines. While, at the highest concentration DT2, increased ROS production by 349% for the HT-29 cell line, while only 193% for HCT-116 cells in comparison to the untreated control cells. This difference in value is due to the lower number of cells due to cytotoxicity. Thymol did not produce any increase in ROS in studied cell lines.

## 3. Discussion

The incidence of colorectal cancer in the world is growing every year. Its treatment is based on surgical removal of the damaged area, or on radiotherapy, immunotherapy, or chemotherapy. A new approach to treatment and prevention may be based on the substances of natural origin, such as thymol, which have the potential to contribute to the understanding of the biological aspects of various cancers, including colorectal cancer. According to the US FDA, thymol is characterized as a safe substance and its use is not limited. Many studies on tumor cell lines have confirmed its toxicity, but the wider use of thymol is limited by its low water solubility. The exact mechanism of penetration of hydrophobic drugs across the cell membrane is unknown. For example, in the case of paclitaxel, its use is also limited due to its lower water solubility [14]. The applied drug concentration is also an important factor. At lower doses, not enough drug may penetrate the membrane. However, at higher doses, it can be toxic. Our newly synthesized and tested derivatives are characterized by increased hydrophilicity, which predicted better penetration of the substance into the cell and increased efficiency at lower concentrations. It was necessary to determine the cytotoxic, genotoxic effect of derivatives, the effect on tumor cell proliferation, micronucleus formation, and ROS production and compare the results with a standard substance.

Thyme essential oil and thymol have a cytotoxic effect on MCF-7 breast cancer cells [15], HepG2 hepatic cancer cells, Caco-2 colon cancer cells, HeLa cervical cancer cells [16], HNSCC cells of squamous cell carcinoma of the oral cavity [17], or inhibit proliferation in N2a neuroblastoma cells [18]. In addition, many studies describe the cytotoxic effect of various synthetic derivatives on colorectal cancer cells [19,20]. In this study, we determined the cytotoxicity of thymol and its derivatives by the MTT assay. This method is suitable to measure cellular metabolic activity as an indicator of cell viability and/or cytotoxicity. Studied compounds affected the cell viability of both colorectal cancer cell lines cultured in vitro in a concentration-dependent manner (Figure 2). The IC_50_ value of thymol (standard) was 52 μg/mL for HT-29 and 65 μg/mL for the HCT-116 cell line. Moreover, the MTT assay indicated that DT1 displayed an approximately 750-fold higher inhibitory effect on the viability of both colorectal cancer cells, in comparison with the thymol-standard. On the other side, the second studied derivative (DT2) had a cytotoxic effect at very high concentrations (1300 μg/mL for HT-29, 2100 μg/mL for HCT-116) compared to the DT1. This large difference in concentrations between DT1 and DT2 is probably due to the chemical structure. Derivative DT2 contains glucoside, and because it is a large molecule, it does not pass so easily into the cells.

In addition to the cytotoxic activity, proliferation activity was also determined for the studied compounds (Figure 1) on both colorectal cancer cell lines after 100 h. The result showed that the HT-29 cell line is more sensitive to the studied compounds than the HCT-116 cell line.

According to a study by Al-Menhali, *Thymus vulgaris* extract affects colorectal cancer cells. It reduces the viability of HCT-116 tumor cells after 24 h of treatment and increases the activity of caspases 3 and 7. The extract contained a high proportion of thymol, carvacrol, and other bioactive ingredients (such as rosmarinic acid or luteolin). An effect on cell invasiveness and migration is also expected [21].

The results were confirmed by a study performed directly with thymol on HCT-116 and LoVo colon tumor cells in 2020 [11]. After a 48 h effect of thymol, the IC_50_ value for HCT-116 was determined to be 47 μg/mL and for LoVo cells 41 μg/mL. Thymol significantly reduced cell migration and invasiveness at a concentration of 40 μg/mL and also had a significant effect on cell colony formation when it reduced cell numbers. No cytotoxic effect was observed on healthy intestinal epithelial cells FHC [11].

These results correlate with our findings. Certain variations in the results could be due to the use of a different method and also to the preparation of the study substance. Zeng et al. isolated thymol from *Thymus vulgaris* extract, while in our study we worked with a commercially purchased substance.

The IC_50_ values determined for the HT-29 cell line according to the available studies are not uniform, but consistently confirm the cytotoxic effect of thymol. According to a 2017 study, the IC_50_ value is ~150 μg/mL after 24 h of thymol treatment. It was the most toxic compound of all the tested substances, the components of essential oils [9]. Cytotoxicity was also monitored in another study by the MTT method and determined to be ~300 μg/mL [22]. Slightly different results may be due to modifications in culture conditions, media, or procedures.

The genotoxic effect was determined by comet and micronucleus assay. In the case of newly synthesized derivatives, we observed a significant genotoxic effect for both cell lines. DT1 reported significantly increased DNA damage in HT-29 and HCT-116 cells even at non-cytotoxic concentrations. Newly synthesized derivative DT1 caused a higher level of DNA damage by 50–60% in the HCT-116 cell line compared to the HT-29 cell line. Derivative of thymol DT2 showed significantly increased DNA damage in both cell lines at a non-cytotoxic concentration of 1000 μg/mL. The thymol-standard did not show a significant genotoxic effect even at the highest tested cytotoxic concentrations in both cell lines. In accordance with the published study [22], neither of our results showed genotoxicity of thymol-standard on HT-29 cells. For the HCT-116 cell line, no study has been published focusing on the genotoxic effect of thymol. However, the induction of single-stranded DNA breaks was not observed in either Caco-2 colon cancer cells or HepG2 human hepatoma cells after 24 h of treatment [23]. Rather, the studies have shown a genoprotective effect of thymol [22] and other essential oils on tumor cell lines HepG2 [24] and colon cell lines HCT15, CO115 [25], and Caco-2 [26].

The micronucleus test did not show an increased micronucleus formation in any of the tested cell lines or in any of the tested substances. There was also no significant increase in apoptotic and mitotic cells. Likewise, carvacrol, an isomer of thymol, does not induce micronucleus formation in immature erythrocyte cells in rats [27].

Cancer cells generate more ROS due to the elevated metabolic process, while increased expression of the antioxidant system supports their survival and this stands as a major cause of drug resistance [28,29]. Therefore, it has been proposed that agents that can cause the elevation of ROS by disturbing the balance of the inbuilt redox system could serve as a potential and safe drug [30]. In this study, to test the level of derivatives of thymol-induced ROS generation, we used ROS-Glo™ H_2_O_2_ Assay. Derivatives of thymol produced ROS at cyto/genotoxic concentrations, which based on our results cause DNA damage. Derivative DT1 significantly increased ROS production only at the highest concentration of 0.08 μg/mL for both studied cell lines. While derivative DT2 from concentration higher than 1000 μg/mL for HCT-116 cell line and 1500 μg/mL for HT-29. The effect of thymol in gastric carcinoma cells (AGS) was evidenced by the elevation of ROS, mitochondrial membrane depolarization, and activation of apoptotic related proteins [31]. Oxidative stress activation was also observed in thymol-treated B16 melanoma cells and non-small lung cancer (A549) cells [32,33]. This confirmed that ROS-mediated toxicity is the principal cancer-killing mechanism of thymol. Moreover, thymol exhibits its pro-oxidant potential when its concentration is increased [33].

## 4. Materials and Methods

### 4.1. Cell Culture

Colorectal carcinoma cell lines HT-29 and HCT-116 were obtained from the American Type Culture Collection (USA). Cells were cultured in Dulbecco’s Modified Eagle Medium (DMEM) in low glucose (1 g/L with added 10% fetal bovine serum (FBS) and 1% penicillin-streptomycin (Thermo Fisher Scientific, Waltham, MA, USA). The cells were placed in an incubator at 37 °C and 5% CO_2_. Media and chemicals used for cell cultivation were purchased from Gibco BRL (Paisley, UK).

### 4.2. Chemicals

**Figure 5:**
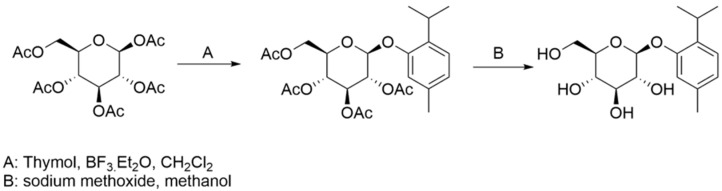
Synthesis of thymol derivatives.

Thymol (purity 99.4%) (T), used as a standard, was obtained from Sigma-Aldrich (St. Louis, MO, USA), acetic acid thymol ester (purity 99.2%) (DT1) was ordered from SynthCluster (Modra, Slovakia). New thymol derivative thymol β-D-glucoside (DT2) was synthesized at the Department of Analytical Chemistry, Faculty of Natural Sciences of Comenius University according to Figure 5.

A: 1,2,3,4,6-penta-O-Ac-β-D-glucose (10 g, 0.025 mol) was dissolved in 100 mL of dichloromethane. Thymol (5 g, 0.033 mol) was added to this solution. After that, BF_3_Et_2_O (4 mL, 0.033 mol) was added dropwise so the temperature did not exceed 25 °C. The reaction mixture was stirred overnight at laboratory temperature. After that, the reaction mixture was neutralized with a 5% Na_2_CO_3_ solution. The organic layer was separated and the water layer was extracted with 2 × 50 mL dichloromethane. Combined organic extracts were washed with water. The solvent was removed by vacuum evaporation and the product was separated by column chromatography with a purity of 98.6%. The product was crystallized from the TBME/ihexane mixture. The yield of the product was 52% (6.5 g).

^1^H NMR (600 MHz, CDCl3) δ: 7.12 (d, *J* = 7.8 Hz, 1H), 7.07 (s, 1H), 6.90 (d, *J* = 7.8 Hz, 1H), 5.65 (d, *J* = 5.2 Hz, 1H), 5.21 (t, *J* = 2.9 Hz, 1H), 4.89 (dd, *J* = 9.6, 2.8 Hz, 1H), 4.25–4.19 (m, 3H), 3.99–3.94 (m, 1H), 3.38–3.25 (m, 1H), 2.30 (s, 3H), 2.13–2.07 (m, 9H), 1.80 (s, 3H), 1.17 (d, *J* = 7.0, 3H), 1.16 (d, *J* = 7.0, 3H) (ESI Figure S1).

^13^C NMR (151 MHz, CDCl3) δ: 170.70, 169.64, 169.14, 150.08, 138.19, 136.20, 126.28, 125.09, 122.63, 121.51, 97.03, 73.30, 70.04, 68.08, 67.14, 63.09, 26.40, 23.40, 23.17, 21.84, 21.01, 20.80, 20.76 (ESI Figure S2).

B: 1-O-thymol-2,3,4,6-tetra-O-Ac-β-D-glucoside (4.6 g, 0.009 mol) was suspended in 50 mL of methanol. 0.5 mL of sodium methoxide solution in methanol (25 wt. % in methanol) was added to the suspension so the pH was maintained in the 8–9 range. The reaction mixture was stirred for 1 h at laboratory temperature. The reaction mixture is neutralized with cathex. The solvent was removed by vacuum evaporation. The yield of the product was 83% (2.5 g).

^1^H NMR (600 MHz, CDCl3) δ: 7.09 (d, *J* = 7.8 Hz, 1H), 6.83 (d, *J* = 7.8 Hz, 1H), 6.79 (s, 1H), 4.84 (d, *J* = 7.2 Hz, 1H), 4.59 (s, 1H), 4.29 (s, 1H), 3.92–3.83 (m, 2H), 3.83–3.66 (m, 3H), 3.42 (dt, *J* = 9.5, 3.5 Hz, 1H), 3.31 (p, *J* = 6.9 Hz, 1H), 2.79 (s, 1H), 2.25 (s, 3H), 1.83 (s, 1H), 1.15 (d, *J* = 7.0, 3H), 1.14 (d, *J* = 7.0, 3H) (ESI Figure S3).

^13^C NMR (151 MHz, CDCl3) δ: 154.03, 136.55, 135.36, 126.10, 124.03, 116.45, 101.64, 76.32, 75.45, 73.63, 69.71, 61.82, 26.08, 23.12, 23.01, 21.18 (ESI Figure S4).

### 4.3. Determination of Cell Proliferation

The rate of cell proliferation was monitored using an IncuCyte ZOOM (Sartorius AG, Göttingen, Germany) [34]. It is a device for displaying a system of living cells in real-time. Cells were seeded in a 96-well plate (2 × 10^4^ cells/well) and the next day treated with the test substance T (0–70 µg/mL), DT1 (0–0.1 µg/mL), or DT2 (0–1000 µg/mL). Immediately after the addition of a substance, the plate was placed in an IncuCyte ZOOM for 100 h with imaging every ten hours. The system determines the percentage confluence for each well.

### 4.4. Determination of Cytotoxicity (MTT Assay)

The cytotoxicity of studied compounds was determined by MTT assay (3-(4,5-dimethyl-thiazolyl)-2,5-diphenyltetrazolium bromide) [35]. Briefly, 2 × 10^4^ cells were seeded in 96-well plates and cultured in a complete DMEM medium. The studied T (0–150 µg/mL), DT1 (0–0.1 µg/mL), DT2 (0–2500 µg/mL) were then added, and the cells were incubated at 37 °C in a 5% CO_2_ atmosphere for 24 h. Next, the samples were washed with phosphate-buffered saline (PBS) at the indicated time point, followed by incubation with 1 mg/mL of MTT for 4 h. Then, the MTT was removed and the formazan crystals were dissolved with dimethyl sulfoxide for 30 min. Absorbance at a wavelength of 540 nm was measured using an xMark microplate spectrophotometer (Bio-Rad Laboratories, Inc., Hercules, CA, USA), and background absorbance at 690 nm was subtracted.

### 4.5. Determination of Genotoxicity (Comet Assay, SCGE)

Genotoxicity was determined by the alkaline comet assay (single-cell gel electrophoresis, SCGE), which allows the detection of DNA breaks [36,37]. Cells were seeded in a 6-well plate (3 × 10^4^ cells/well) and treated with a tested substance for 24 h. Cells were embedded in 0.75% LMP agarose (low melting point) and plated on a slide coated with NMP agarose (normal melting point). Lysis was performed in a cooled solution consisting of 2.5 mol/L NaCl, 100 mmol/L Na_2_EDTA, 10 mmol/L Tri-HCl (pH 10), and 1% Triton X-100 for one hour in the cold. The samples were transferred to an electrophoretic solution (300 mmol/L NaOH, 1 mmol/L Na_2_EDTA, pH > 13) in an electrophoretic apparatus and allowed to unwind for 30 min at 4 °C in the dark. Electrophoresis (19 V, 300 mA) was then performed for 20 min at 4 °C. Samples were neutralized in neutralization solution 2 × 10 min (0.4 mol/L Tris-HCl, pH 7.4) and fixed in ethanol for 5 min. After the slides had dried, ethidium bromide (5 µg/mL) was applied. Slides were examined with Zeiss Imager.Z2 fluorescence microscope using the computerized image analysis (Metafer 3.6, MetaSystems GmbH, Altlussheim, Germany). The percentage of DNA in the tail (% of tail DNA) was used as a parameter for the measurement of DNA damage (DNA strand breaks). One hundred comets were scored per sample in one electrophoresis run.

### 4.6. Determination of Genotoxicity (Micronucleus Assay)

The genotoxic effect was also assessed by a micronucleus test described by Ujhazy et al. [38]. The cells were seeded in 60 mm Petri dishes (4 × 10^5^). Cells were treated after 24 h of growth (60 µg/mL for T; 0.08 µg/mL for DT1; 2000 µg/mL for DT2) and allowed to proliferate for another 24 h. The cells were fixed with an ice-cold fixative solution (methanol and acetic acid 1:3) for 15 min, washed with distilled water, and allowed to dry. The next day, they were stained with a solution of DAPI (0.2 μg/mL) and McIlvaine (0.2 mol/L Na_2_HPO_4_) for 40 min. They were then washed with McIlvaine solution for 3 min, washed with distilled water, and allowed to dry overnight. Prior to evaluation, 2 × 40 μL of glycerol was applied to each plate. Samples were evaluated with an Olympus BX51 fluorescence microscope, with 2000 cells per plate in four categories: interphase, apoptosis and necrosis, micronucleus, and mitosis.

### 4.7. Determination of ROS Production

The oxidative stress was analyzed using the ROS-Glo™ H_2_O_2_ Assay (Promega, Madison, WI, USA) according to the manufacturer’s protocol. The ROS-Glo™ H_2_O_2_ Assay is a bioluminescent assay that measures the level of hydrogen peroxide (H_2_O_2_), a reactive oxygen species (ROS), directly in cell culture or in defined enzyme reactions. Briefly, 2 × 10^4^ cells were seeded in 96-well plates and cultured in a complete DMEM medium. The studied T (10–60 µg/mL), DT1 (0.01–0.08 µg/mL), DT2 (500–2000 µg/mL) were then added, and the cells were incubated at 37 °C in a 5% CO_2_ atmosphere for 24 h. H_2_O_2_ substrate solution was added for 6 h to generate a luciferin precursor. The addition of ROS-Glo™ Detection Solution (20 min) converts the precursor to luciferin and provides Ultra-Glo™ Recombinant Luciferase to produce a light signal that is proportional to the level of H_2_O_2_ present in the sample. The relative luminescence was measured using a GloMax^®®^ Discover Microplate Reader.

### 4.8. Statistical Analysis

The results represent a mean from 3 to 5 experiments ± standard deviation (SD). The differences between defined groups were tested for statistical significance using Student’s *t*-test (* *p* < 0.05; ** *p* < 0.01; *** *p* < 0.001).

## 5. Conclusions

Thymol has a cytotoxic effect on colorectal cancer tumor cells HT-29 and HCT-116. However, the effects of newly synthesized thymol derivatives on cells depend on the chemical structure. In this study, the derivative of thymol, acetic acid thymol ester shows cyto/genotoxic effects and produces ROS at much lower concentrations (~0.08 µg/mL) than the standard (~60 μg/mL). This confirmed the hypothesis that the hydrophilic properties of the derivatives have the potential to act more effectively and have the potential to be applied in the treatment or prevention of colorectal cancer. However, further and more detailed research on these derivatives is needed in the future.

## Figures and Tables

**Table 1:** Effect of thymol (T), acetic acid thymol ester (DT1), and thymol β-D-glucoside (DT2) after 24 h on ROS production in HT-29 and HCT-116 cell lines.

Concentrations (μg/mL)/Cell Lines	HT-29	HCT-116
Negative control	1,52 × 10^4^ ± 3845.1	4.1 × 10^4^ ± 4619.5
Positive control	2.9 × 10^5^ ± 17975.3 ***	1.5 × 10^6^ ± 19035.1 ***
T 10	9.2 × 10^3^ ± 105.3	3.7 × 10^4^ ± 5288.5
T 25	9.8 × 10^3^ ± 504.5	3.3 × 10^4^ ± 6778.1
T 50	9.6 × 10^3^ ± 2076.1	3.1 × 10^4^ ± 2256.6
T 60	8.7 × 10^3^ ± 1039.4	3.0 × 10^4^ ± 7231.1
DT1 0.01	1.3 × 10^4^ ± 1406.6	4.2 × 10^4^ ± 195.8
DT1 0.02	1.5 × 10^4^ ± 1162.2	5.2 × 10^4^ ± 478.0
DT1 0.04	2.3 × 10^4^ ± 6162.2	4.9 × 10^4^ ± 4555.1
DT1 0.08	5.8 × 10^4^ ± 7748.5 **	6.2 × 10^4^ ± 7018.2 *
DT2 500	1.2 × 10^4^ ± 2164.7	3.9 × 10^4^ ± 1952.8
DT2 1000	1.6 × 10^4^ ± 1037.7	7.3 × 10^4^ ± 6570.4 **
DT2 1500	3.2 × 10^4^ ± 5494.4 *	9.4 × 10^4^ ± 4602.8 ***
DT3 2000	5.3 × 10^4^ ± 5360.1 **	7.9 × 10^4^ ± 3002.5 ***

Positive control-menadione (50 μmol/L). Data represent means ± SD of three in-dependent experiments. * *p* < 0.05; ** *p* < 0.01; *** *p* < 0.001 indicate statistically significant differences compared to the control (Student’s *t*-test).

## Data Availability

Data is contained within the article.

## Supplementary Materials

The following supporting information can be downloaded at: https://www.mdpi.com/article/10.3390/molecules27092622/s1. Figure S1: 1H NMR (600 MHz) spectrum of 1-O-thymol-2,3,4,6-tetra-O-Ac-β-D-glucoside in CDCl3; Figure S2: 13C NMR (151 MHz) spectrum of 1-O-thymol-2,3,4,6-tetra-O-Ac-β-D-glucoside in CDCl3; Figure S3: 1H NMR (600 MHz) spectrum of thymol-β-D-glucoside in CDCl3; Figure S4: 13C NMR (151 MHz) spectrum of thymol-β-D-glucoside in CDCl3.
