# Supplementary material for: Newly Synthesized Thymol Derivative and Its Effect on Colorectal Cancer Cells
Source: Molecules. 2022 Apr 19;27(9):2622. doi: 10.3390/molecules27092622 (PMC9103784; doi:10.3390/molecules27092622)
Supplement: Supplementary file 1 [file molecules-27-02622-s001.zip › molecules-1683876-supplementary.pdf]

Supplementary material:

Newly synthesized thymol derivatives and their effect on colo-rectal cancer cells

Michaela Blažíčková[1], Jaroslav Blaško[2], Róbert Kubinec[2], Katarína Kozics[1,*]

[1] Cancer Research Institute, Biomedical Research Center, Slovak Academy of Sciences, Dúbravská cesta 9, 84505 Bratislava, Slovakia; katarina.kozics@savba.sk
[2] Department of Analytical Chemistry, Faculty of Natural Sciences, Comenius University in Bratislava, Mlynská dolina, Ilkovičova 6, 84215 Bratislava, Slovakia; robert.kubinec@uniba.sk
* Correspondence: katarina.kozics@savba.sk; Tel.: +421-232295176

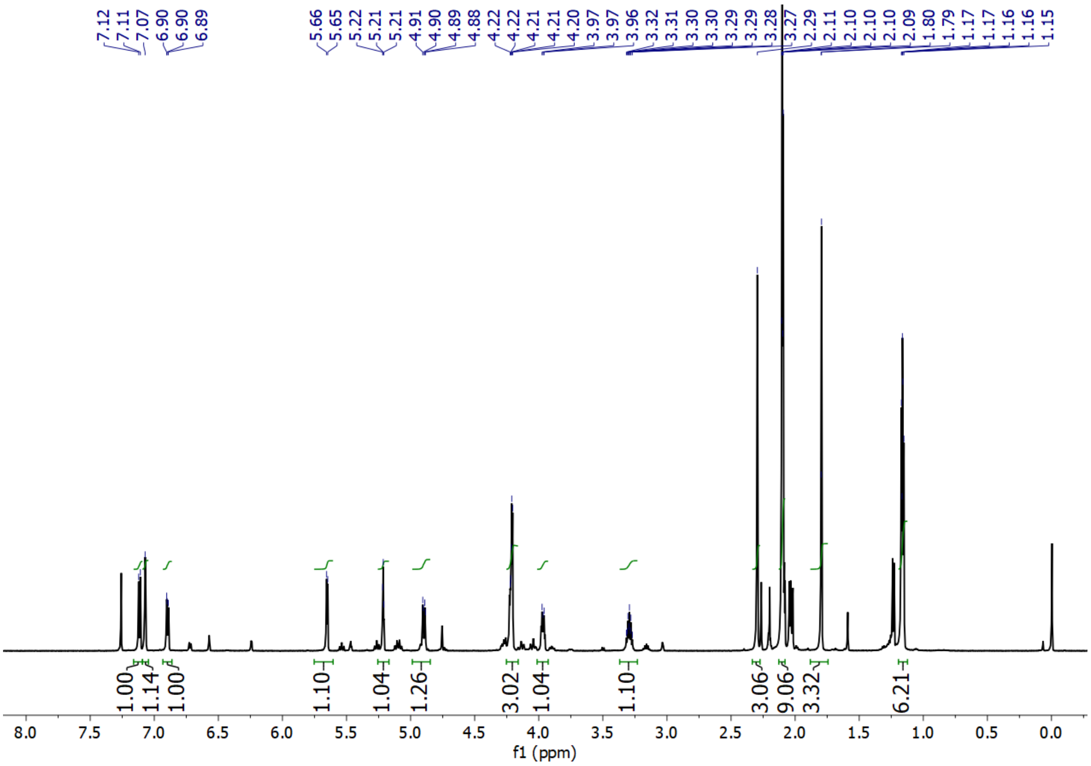

Figure S1. ^{1}H NMR (600 MHz) spectrum of 1-O-thymol-2,3,4,6-tetra-O-Ac-β-D-glucoside in $CDCl_3$.

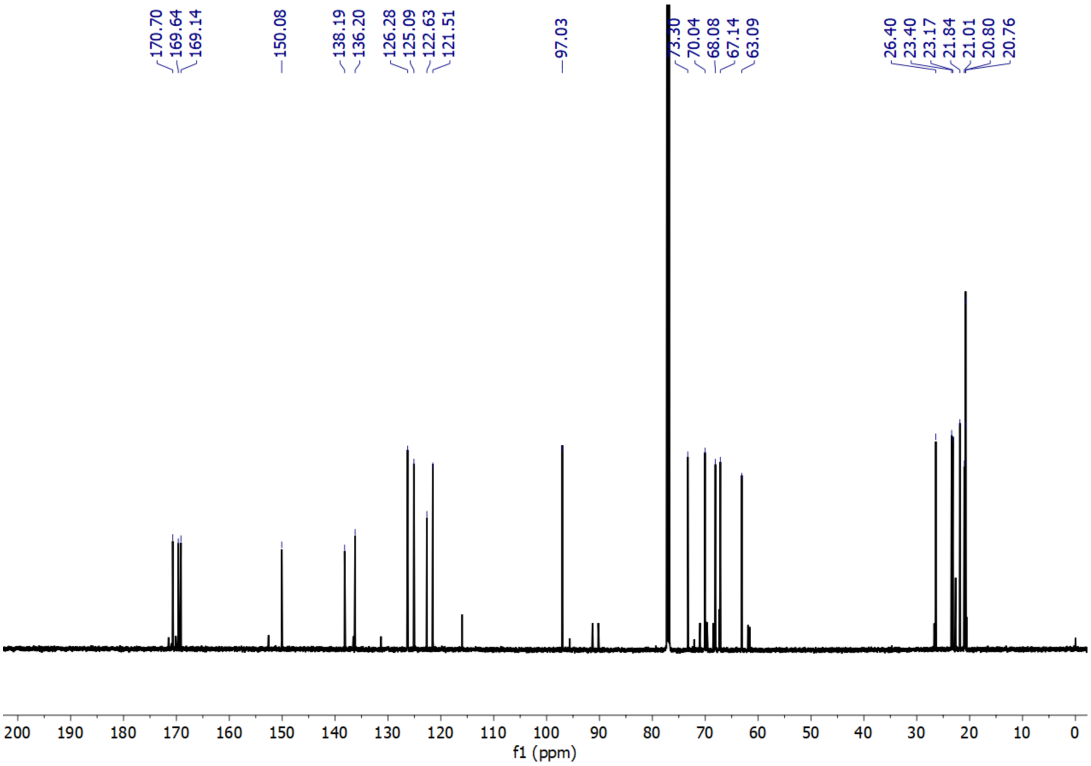

Figure S2. ^{13}C NMR (151 MHz) spectrum of 1-O-thymol-2,3,4,6-tetra-O-Ac-β-D-glucoside in $CDCl_3$.

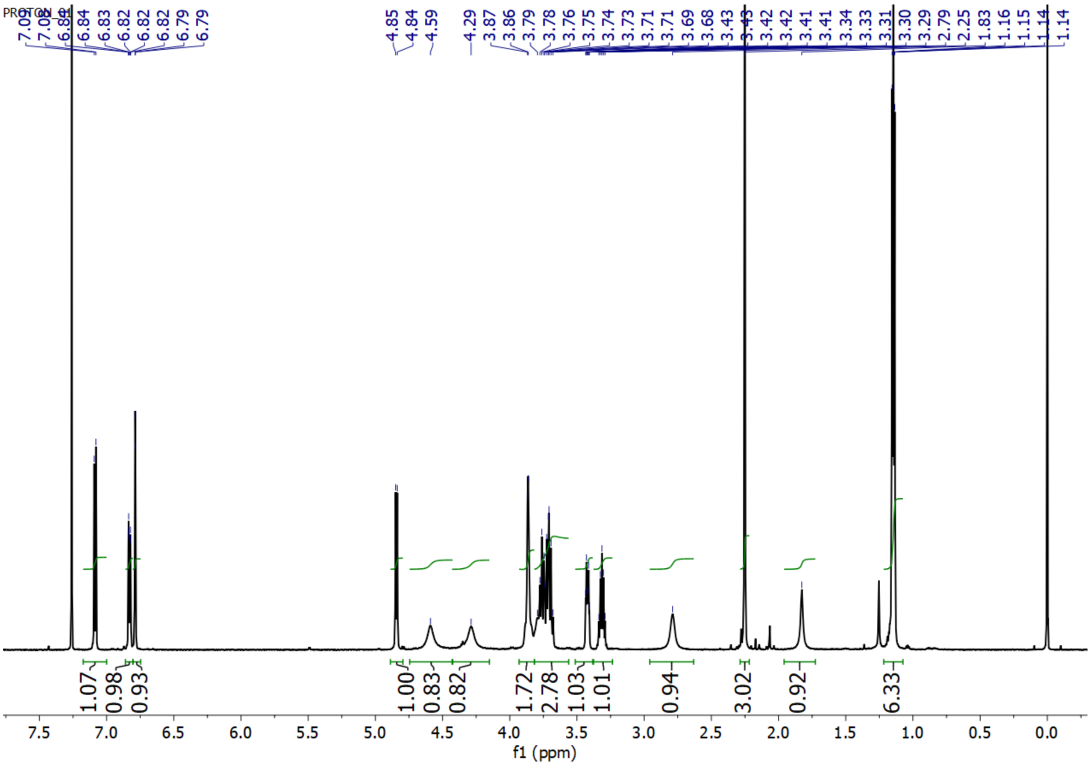

Figure S3. ^{1}H NMR (600 MHz) spectrum of thymol-β-D-glucoside in $CDCl_3$.

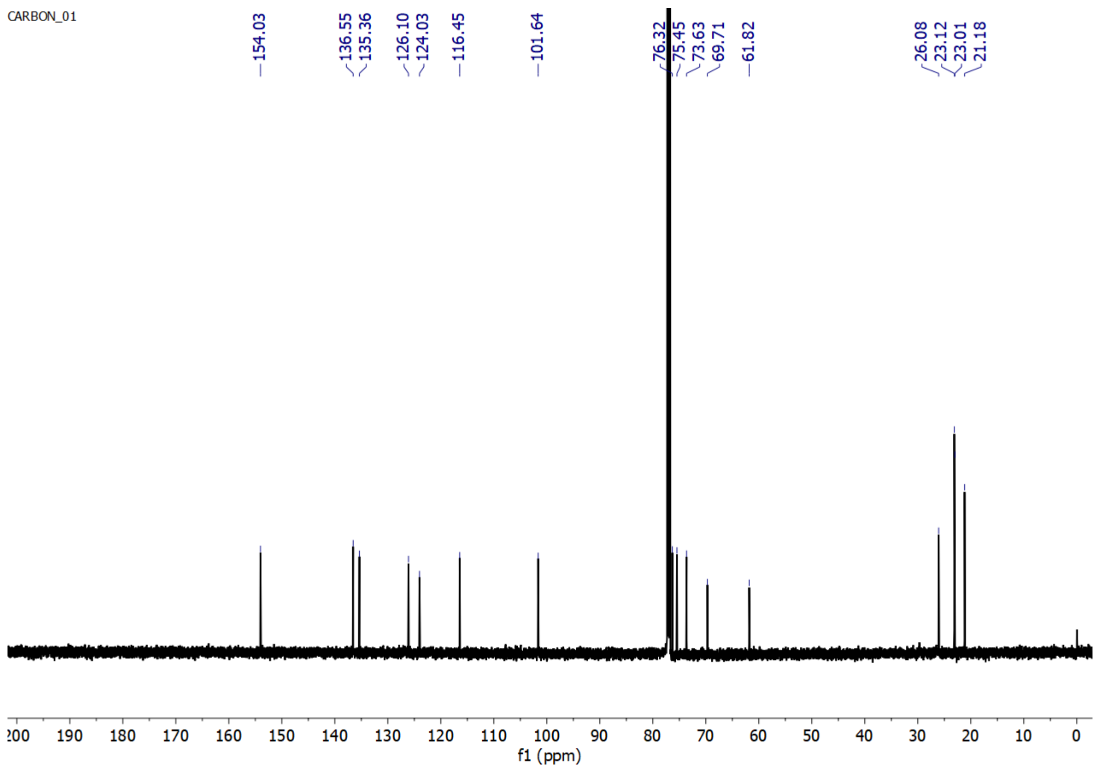

Figure S4. ^{13}C NMR (151 MHz) spectrum of thymol-β-D-glucoside in $CDCl_3$.
